# Mortality and clinical characteristics of multisystem inflammatory syndrome in children (MIS-C) associated with covid-19 in critically ill patients: an observational multicenter study (MISCO study)

**DOI:** 10.1186/s12887-021-02974-9

**Published:** 2021-11-18

**Authors:** Lorena Acevedo, Byron Enrique Piñeres-Olave, Laura Fernanda Niño-Serna, Liliana Mazzillo Vega, Ivan Jose Ardila Gomez, Shayl Chacón, Juan Camilo Jaramillo-Bustamante, Hernando Mulett-Hoyos, Otto González-Pardo, Eliana Zemanate, Ledys Izquierdo, Jaime Piracoca Mejìa, Jose Luis Junco González, Beatriz Giraldo Duran, Carolina Bonilla Gonzalez, Helen Preciado, Rafael Orozco Marun, Martha I Alvarez-Olmos, Carolina Giraldo Alzate, Jorge Rojas, Juan Carlos Salazar-Uribe, Juan-Manuel Anaya, Jaime Fernández-Sarmiento

**Affiliations:** 1 Department of Pediatrics and Intensive Care. Fundación Cardioinfantil-Instituto de Cardiología, Universidad de la Sabana, Bogotá, Colombia; 2 Department of Pediatrics and Intensive Care. Clínica CardioVID, Medellín, Colombia; 3 Department of Pediatrics. Hospital Pablo Tobón Uribe, Universidad de Antioquia, Medellín, Colombia; 4 Department of Pediatrics and Intensive Care, Hospital Infantil Los Ángeles, Pasto, Colombia; 5 Department of Pediatrics and Intensive Care. Clínica Uros, Neiva, Colombia; 6 Department of Pediatrics and Intensive Care, Hospital Universitario San Ignacio, Bogotá, Colombia; 7 Department of Pediatrics and Intensive Care, Hospital General de Medellín, Universidad de Antioquia, Red Colaborativa Pediátrica de Latinoamérica (LARed Network, Medellín, Colombia; 8 Department of Pediatrics and Intensive Care, Fundación Cardioinfantil-Instituto de Cardiología, Universidad del Rosario, Bogotá, Colombia; 9 Department of Pediatrics and Intensive Care, Fundación Clínica Shaio, Universidad Nacional de Colombia, Bogotá, Colombia; 10 Department of Pediatrics and Intensive Care, Hospital Susana Lopez de Valencia, Universidad del Cauca, Popayán, Colombia; 11 Department of Pediatrics and Intensive Care, Hospital Militar Central, Hospital Santa Clara, Bogotá, Colombia; 12 Department of Pediatrics and Intensive Care, Clínica Infantil de Colsubsidio, Bogotá, Colombia; 13 Department of Pediatrics and Intensive Care, Instituto Roosevelt, Bogotá, Colombia; 14 Department of Pediatrics and Intensive Care, Hospital Infantil de la Cruz Roja Rafael Henao Toro, Manizales, Colombia; 15 Department of Pediatrics and Intensive Care, Fundación Santa Fe de Bogotá, Bogotá, Colombia; 16 Department of Pediatrics and Intensive Care, Fundación Universitaria de Ciencias de la Salud. Hospital de San José, Bogotá, Colombia; 17 Department of Pediatrics and Intensive Care, Clínica Portoazul, Puerto Colombia, Colombia; 18 Department of Pediatrics and Infectious Diseases, Fundación Cardioinfantil-Instituto de Cardiología, Universidad del Rosario, Bogotá, Colombia; 19 Department of Pediatrics and Intensive Care, Hospital Pablo Tobón Uribe, Medellín, Colombia; 20 Department of Pediatrics and Intensive Care, Hospital Santa Clara, Bogotá, Colombia; 21 Research Group in Statistics, Universidad Nacional de Colombia, Medellín, Colombia; 22 Center for Autoimmune Disease Research (CREA), School of Medicine and Health Sciences, Universidad del Rosario, Bogotá, Colombia; 23 Universidad CES Graduate School, Medellín, Colombia; 24 Universidad de La Sabana, Campus Universitario del Puente del Común, Km 7 Autopista Norte de Bogotá, Cundinamarca Chía, Colombia

**Keywords:** Sepsis, SARS-CoV2, PIMS-TS, inflammatory, mortality

## Abstract

**Background:**

The clinical presentation and severity of Multisystem Inflammatory Syndrome in Children associated with COVID-19 (MIS-C) is widespread and presents a very low mortality rate in high-income countries. This research describes the clinical characteristics of MIS-C in critically ill children in middle-income countries and the factors associated with the rate of mortality and patients with critical outcomes.

**Methods:**

An observational cohort study was conducted in 14 pediatric intensive care units (PICUs) in Colombia between April 01, 2020, and January 31, 2021. Patient age ranged between one month and 18 years, and each patient met the requirements set forth by the World Health Organization (WHO) for MIS-C.

**Results:**

There were seventy-eight children in this study. The median age was seven years (IQR 1-11), 18 % (14/78) were under one year old, and 56 % were male. 35 % of patients (29/78) were obese or overweight. The PICU stay per individual was six days (IQR 4-7), and 100 % had a fever upon arrival to the clinic lasting at least five days (IQR 3.7-6). 70 % (55/78) of patients had diarrhea, and 87 % (68/78) had shock or systolic myocardial dysfunction (78 %). Coronary aneurysms were found in 35 % (27/78) of cases, and pericardial effusion was found in 36 %. When compared to existing data in high-income countries, there was a higher mortality rate observed (9 % vs. 1.8 %; p=0.001). When assessing the group of patients that did not survive, a higher frequency of ferritin levels was found, above 500 ngr/mL (100 % vs. 45 %; p=0.012), as well as more cardiovascular complications (100 % vs. 54 %; p = 0.019) when compared to the group that survived. The main treatments received were immunoglobulin (91 %), vasoactive support (76 %), steroids (70.5 %) and antiplatelets (44 %).

**Conclusions:**

Multisystem Inflammatory Syndrome in Children due to SARS-CoV-2 in critically ill children living in a middle-income country has some clinical, laboratory, and echocardiographic characteristics similar to those described in high-income countries. The observed inflammatory response and cardiovascular involvement were conditions that, added to the later presentation, may explain the higher mortality seen in these children.

**Supplementary Information:**

The online version contains supplementary material available at 10.1186/s12887-021-02974-9.

## Background

Since December 2019, when severe acute respiratory syndrome coronavirus 2 (SARS-CoV-2) infection was constituted as a global health threat, high morbidity and mortality have been seen worldwide [1]. This virus, which caused the 2019 coronavirus disease (COVID-19), has been characterized by high transmissibility, severity, and lethality in adults [2, 3]. Towards the end of April 2020, the UK and several European nations reported the appearance of a new presentation of SARS-CoV-2 infection, known as “Multisystem Inflammatory Syndrome in Children (MIS-C),” which behaves more aggressively, causing up to ten times greater mortality than that described for COVID-19 in the pediatric population [4, 5].

This clinical behavior is characterized by a severe hyperinflammatory state clinically similar to other diseases such as Kawasaki disease, toxic shock syndrome, and macrophage activation syndrome [6, 7]. The pandemic began in China, spreading to Europe and North America and then to other world regions. Specifically, in May 2020, Latin America became the epicenter of the COVID-19 pandemic, an area already affected by social inequalities, with significant limitations in access to medical care, inadequate nutrition, and a high prevalence of many chronic non-communicable diseases [2].

The peak of MIS-C in most studies has been observed 4-6 weeks after the adult population’s highest impact. With this MIS-C behavior around the globe, we expected to see the first cases in Latin America beginning in July 2020 [8, 9]. Just as expected, cases of MIS-C began to appear around the hypothesized time frame. Given the genetic, social, economic conditions and healthcare access in this region, this syndrome may exhibit different clinical behavior including severity, duration, and sequelae compared to what has been described in other parts of the world [2, 8, 10]. The objective of this study was to describe the characteristics, clinical course and mortality rate of MIS-C in a middle-income country and evaluate if there are differences in the affected groups and outcomes compared to high-income countries.

### Methods

A multicenter observational study with retrospective and prospective components was carried out in 14 PICUs in Colombia from April 01, 2020, to January 31, 2021. The children that were consecutively included for the study met the WHO criteria for MIS-C (Table 1), ranged from ages one month to 18 years of age, and were hospitalized in pediatric intensive care due to the severity of their condition. Additionally, the patients had to have had an RT-PCR, antibody, or antigen test within the first 48 h of admission to intensive care showing current or recent SARS-CoV-2 infection. Patients with incomplete MIS-C criteria, or those with complete criteria for Kawasaki disease or toxic shock syndrome, were excluded. All patients were monitored during their stay in intensive care. Clinical and echocardiographic variables, as well as lab exams and clinical progress, were also monitored.

**Table 1:** Demographic, clinical, laboratory and treatment characteristics of children included in the MISCO Study

Demographic characteristics	n = 78
Age in years, median (IQR)	7 (1 - 11)
Male sex, n (%)	46 (59)
Origin according to altitude, n(%)	
> 1,500 MASL	69 (88.5)
< 1,500 MASL	9 (11.5)
Nutritional status, n (%)	
Obesity	23 (29.5)
Overweight	6 (7.7)
Normal	45 (57.7)
Undernutrition	4 (5.1)
Days in PICU, median (IQR)	6 (4 – 7)
Death, n (%)	7 (9)
Days from onset of symptoms to admission to PICU (IQR)	
Survivors	5 (1-92)
No survivors	6 (3-12)
Clinical characteristics	
Days from onset of symptoms and test, median (IQR)	4 (3 – 5)
Days of fever, median (IQR)	5 (3.75 – 6)
Organ involvement	
Skin and mucous membranes, n (%)	
Lips/tongue	17 (22)
Hand/foot edema	34 (44)
Conjunctival injection	23 (29.5)
Gastrointestinal, n(%)	
Diarrhea	55 (70.5)
Vomiting	58 (74)
Abdominal pain	55 (70.5)
Neurological disorders, n (%)	
Headache	17 (22)
Cardiovascular	
Shock	68 (87)
Hypotension	61 (78)
Cardiac dysfunction ₸	14 (19)
Coronary artery dilation or aneurysms₸	27 (35)
Mitral regurgitation₸	16 (21)
Aortic regurgitation₸	2 (2.5)
Pericardial effusion₸	27 (36)
Arrhythmias	5 (6)
Elevated troponin	38/75 (51)
Elevated proBNP (> 400 pg/m)	27/33 (82)
Renal, n (%)	
Acute kidney injury	23 (29)
Respiratory	
Lower PaO2/FiO2, median (IQR)**	152 (98 - 260)
Higher oxygenation index, median (IQR) ***	14 (8.7 – 25.5)
Hematological, n(%)	
Elevated D-dimer > 3,000 ng/ml	41 (55)
Anemia	36 (46)
Thrombocytopenia^†^	28 (36)
Lymphopenia^*^	50 (64)
Elevated PTT	15 (19)
Elevated PT	8 (10)
Other, n(%)	
Cervical lymphadenopathy > 1.5 cm	18 (23)
SARS COV-2 Labs, n (%)	
Positive PCR	52 (67)
Positive antigen	1 (1)
Negative PCR/Positive serology	15 (19)
Positive serology	10 (13)
Treatment	
Immunoglobulin	71 (91%)
Immunomodulator (eculizumab)	1 (1%)
Steroids	55 (70.5%)
Antivirals (Lopinavir)	1 (1%)
Antiplatelet drugs	34 (44%)
Anticoagulants	34 (44%)
Vasoactive drugs, n (%)	59 (76%)
Days of vasoactive drug use, median (IQR)	3 (2-5)
Vasoactive -Inotropic score(VIS), median (IQR)	21 (10 – 35)
Respiratory support¶	
HFNC	24 (31%)
NIV	3 (4%)
Conventional MV	22 (28%)
HFOV	5 (6.4%)
Dialysis (RRT)	9 (11.5%)

₸ In 75 patients who had an echocardiogram. * Lymphopenia less than 4500 cells /uL. ** Lower Pa02/Fi02 during PICU stay. ***Higher oxygenation index (OI) during PICU stay. PT – prothrombin time. PTT – partial thromboplastin time. HFNC – high-flow nasal cannula. NIV – noninvasive ventilation. MV – mechanical ventilation. HFOV – high-frequency oscillatory ventilation. RRT – renal replacement therapy. MASL --meters above sea level. ¶ A single patient may have had more than one of the described respiratory supports during his/her PICU stay

 This study was approved by an ethics committee at each participating institution, which was a requirement for including patients. The information was taken from the patients’ charts and recorded in a database designed specifically for this study, to which only the principal investigators had access. All the data of interest was recorded in the database, and patients with incomplete data were not included. Demographics, clinical characteristics, general acute phase reactants and those related to severity were recorded. The involvement of the different organs was assessed through clinical and paraclinical data. Likewise, the cardiovascular involvement was recorded by taking into account vasopressor support, troponin and proBNP level, and the recording of echocardiograms performed, including measurement of the abnormalities found and the size of coronary aneurysms, using the Z-score. The echocardiogram was performed within 48 h of admission to the PICU by a cardiologist with at least five years of experience. In some middle and low-income country hospitals, a cardiologist is not readily available to perform an echocardiogram within 24 h of admission.

### Definitions

The WHO definition of MIS-C was used, which includes age (0-19 years) and two of the following findings: (a) a rash, bilateral non-purulent conjunctivitis or mucocutaneous inflammation signs (oral, hands, feet); (b) hypotension or shock; (c) signs of myocardial dysfunction, pericarditis, valvulitis or coronary abnormalities (includes echocardiographic findings or elevated troponins/high proBNP); (d) evidence of coagulopathy (elevated D-dimer, PT, PTT); (e) acute gastrointestinal symptoms (diarrhea, vomiting, or abdominal pain) and elevated inflammatory markers (ESR, CRP, or PCT) AND absence of other microbial causes of inflammation (bacterial sepsis, staphylococcal or streptococcal TSS) AND evidence of SARS-CoV-2 infection (RT-PCR, antigen test, positive serology) or contact with a COVID-19 patient. We defined coronary dilation as a Z-score between 2.0 and 2.4 and coronary aneurysm as a Z-score greater than 2.5. If the patient had more than one echocardiogram and follow up, all of the data was recorded. Systolic myocardial dysfunction was considered to be an ejection fraction less than 60 %. Mechanical ventilation support and vasopressor requirement were recorded using the vasoactive and inotropic score (VIS) [11] [dopamine (mcg/kg/min) + dobutamine (mcg/kg/min) +10 x milrinone (mcg/kg/min) + 100 x adrenaline (mcg/kg/min) + 100 x noradrenaline (mcg/kg/min) + 10,000 x vasopressin (U/kg/min)], along with the treatments received in both dose and frequency. The acute kidney injury severity scale was recorded using KDIGO, as well as the need for renal replacement therapy due to acute kidney injury. Thrombocytopenia was defined as a platelet count less than 150,000, and lymphopenia was defined as a lymphocyte count less than 4,500 in children under eight months old and less than 1,500 in children over eight months old. Overweight children under five years old were defined according to the WHO recommendations for weight and height as more than two standard deviations above the median, and obesity was defined as more than three standard deviations, as established in the WHO child growth standards. Overweight children between the ages of 5-19 were defined as a body mass index (BMI) for age more than one standard deviation, and obesity as more than two standard deviations, above the median established in the WHO child growth standards. The PICU stay was defined as the number of days from admission to transfer to a general hospital bed. Shock was defined as patients with signs of low cardiac output (altered consciousness, capillary refill greater than two seconds, weak pulses or oliguria) with or without hypotension. Hypotension was considered to be systolic arterial pressure under the fifth percentile for age.

### Sample study

The diagnosis of MIS-C according to the WHO criteria included a positive SARS-CoV-2 test such as the reverse transcription-polymerase chain reaction (RT-PCR) test, with samples taken from a nasopharyngeal swab or tracheal aspiration. In cases where an IgM or IgG antibody test (immunodiffusion chromatography – Abbott) or serum antigen test was available, it was recorded.

### Statistical analysis

A descriptive analysis was performed using measures of central tendency according to the distribution of the variable, reporting means with their standard deviations or medians with interquartile ranges. Qualitative variables are reported in frequencies and percentages. A bivariate analysis was performed comparing the results of this study with results of MIS-C cases in the US reported by the CDC in Atlanta between April and October 2020 [8]. Likewise, the results were compared with what was reported by Swann et al. regarding the behavior of MIS-C in the UK [12]. ANOVA was used for comparing means of more than two groups with a non-normal distribution, and Mann-Whitney U for fewer than two groups. Student’s t-test was used for comparing two independent groups with equal variance. These two reports in high-income countries were used, as the authors felt they were the most complete studies with the most representative data. Chi2 or Fisher’s exact test were used for qualitative variables, and the Friedman test for repeated measures was employed for laboratory tests.

To estimate the odds ratios (ORs) and hazard ratios (HRs) of the factors at onset of symptoms that were substantially associated with mortality, we used both a logistic regression model for a dichotomous response and a proportional hazard Cox regression model.

In the first model, a logistic regression model was fitted in order to estimate the effect size of significant variables on mortality which was coded as a dichotomous variable. The dependent variable of the logistic model was the natural log of the odds of survival. The independent variables of the Cox model were selected through a backward selection procedure. To assess the clinical importance of significant variables, odds ratios (ORs) were computed. 95 % confidence intervals (CIs) for ORs and regression coefficients were also calculated. P-values <0.05 were considered significant. Statistical analyses were performed by using SAS PROC LOGISTIC implemented in SAS Studio.

In the second model, the Cox model, allowed us to estimate the effect size of significant variables at onset of symptoms associated with mortality. The dependent variable of the Cox model was the natural log of the risk of death at time t. The independent variables of the Cox model were selected through a backward selection procedure. To assess the clinical importance of significant variables, hazard ratios (HRs) were computed. A hazard ratio measures the effect size of a variable on onset of symptoms to death. 95 % confidence intervals (CIs) for HRs and regression coefficients were also calculated. P-values <0.05 were considered significant. Statistical analyses were performed by using SAS PROC LIFEREG and SAS PROC PHREG implemented in SAS Studio.

## Results

### Clinical characteristics of the patients

**Fig. 1:**
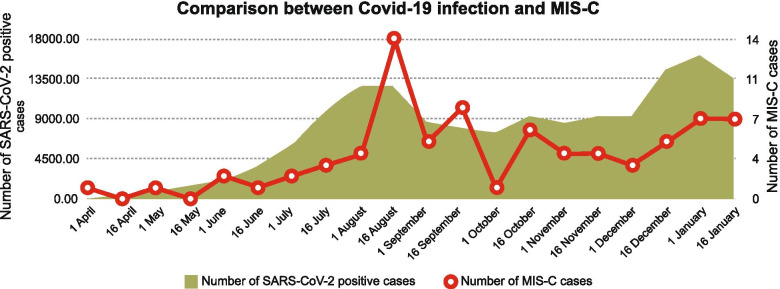
Temporal relation between COVID-19 infections and MIS-C in critically ill children under the age of 18

During the study period from April 2020 to January 31, 2021, 155,207 cases of SARS-CoV-2 infection in children were reported in Colombia, which corresponds to 7.4 % of all adult infections. Of these patients, 0.1 % were hospitalized in pediatric intensive care. Figure 1 shows the relationship between COVID-19 infections in children and MIS-C in critically ill patients under the age of 18 from April 2020 to January 2021.

A total of 78 patients with MIS-C who were admitted to the 14 PICUs participating in this study were included. Fourteen patients (18 %) were under one year old. This age group showed no difference in the frequency of cardiovascular complications (p=0.96) or mortality (p=0.1) compared to older children. Overweight and obesity were found in 29 patients (37 %). The most prevalent symptom observed was fever; all children who were admitted had a fever upon admission lasting for at least 5 days. Frequent secondary symptoms included gastrointestinal (diarrhea, vomiting and abdominal pain) (Table 1). Sixty-eight patients were in shock (87 %) on admission, and 27 children (36 %) had pericardial effusion. The most frequent arrhythmias were supraventricular tachycardia in three, ventricular tachycardia in one and ventricular extrasystoles in another.

### SARS-CoV-2 test results

The diagnosis was made via SARS-CoV-2 RT-PCR in 53 (68 %) patients, an antibody test in 24 (30.1 %), and by antigens in one (Table 1).

### Inflammatory markers

On admission, 62 patients (79 %) had a CRP greater than 3 mg/dL, and of those whose ESR was measured, 50/63 (64 %) were over 40 mm/h. On admission, the lowest D-dimer value was 310 and the highest was 20,000 ng/ml. Ferritin was over 500 in 39 patients (50 %). Additional file 1 shows the behavior of the inflammatory markers and other labs on admission, and in some cases, at 24 and 48 h of follow up.

### Other labs

Lymphopenia was seen in 50 patients (64 %). All those under one year old had an albumin level of less than 2.5 g/dL, and all but one of the patients over the age of one had values less than 3.5 g/dL. Elevated total bilirubin that was atypical for age was found in 25/54 patients (46 %) (Additional file 1). Acute kidney injury was seen in 29 % of cases, 55 % of cases had elevated D-dimer, and anemia was seen in 46 % of cases.

### Echocardiogram results

An echocardiogram was performed on 75 patients (96 %) in which median left ventricular ejection fraction was 65 % (IQR: 57-70). These findings were consistent between patients who survived and those who did not (p=0.5), and the median fractional shortening was 37 % (IQR: 29-40). Coronary anomalies were found in 35 patients (47 %); 27 patients (36 %) had a Z-score greater than 2.5 which was compatible with coronary aneurysms. Fourteen patients only had left coronary involvement, six had right coronary involvement, and six had anterior descending artery involvement. Ten patients had two affected coronary arteries, 16 had coronary artery hyper refringence, four had ectasia, and seven had hyper refringence and ectasia.

### Treatment received

Most of the patients did not receive antiviral treatment (98.7 %). Steroids were given to 55 patients (70.5 %), 42 (54 %) received three to five-day methylprednisolone bursts, and 19 (24 %) received dexamethasone for three to ten days. Seventy-one patients (91 %) received immunoglobulin treatment. Inotropic support was required for 76 % of the patients, with a vasoactive score ranging from 4 in the best case scenario, to 345 in the worst case scenario. Of the 11 patients who received invasive mechanical ventilation, five (6 %) required high-frequency oscillatory ventilation, which included four of the five patients who died. Six patients (8 %) required MV for more than 10 days.

### Outcomes

#### Mortality

There was a 9 % mortality rate (7/78) in our study (Table 2). The group that did not survive had a longer duration of the disease prior to being admitted to the PICU (6 days vs. 5 days; p = 0.003), more frequency of ferritin above 500 ngr/mL (100 % vs. 45 %; p = 0.012), more cardiovascular complications (100 % vs. 54 %; p = 0.019), more need for mechanical ventilation support (100 % vs. 21 %; p = 0.001), and more need for renal replacement therapy (86 % vs. 4.2 %; p < 0.001) compared to the group that survived (Table 2*).* Additionally, the group that did not survive had less diarrhea (14.3 % vs. 76.1 %; OR 0.04 95 % CI 0.001, 0.27;p=0.01) and normal nutritional status (100 % vs. 53.5 %;p=0.018). ). In this way, the arrhythmias (HR 0.03 95 %CI 0.002, 0.40; p=0.008) and pericarditis (HR 0.03 95 % IC 0.003, 0.408;p=0.07) were important variables associated from the onset of symptoms to death (Table 3- Additional file 2). Immunoglobulin (p=0.3) and steroid administration (p=0.4) did not make a difference in regards to survival rate.

**Table 2:** Demographic, clinical and laboratory characteristics of child survivors vs. non-survivors included in the MISCO Study

Demographic characteristics	Survivors n= 71	No**n**-survivors n=7	p-value^**a**^
Age < 7 years, n (%)	32(45.1)	5 (71.4)	0.23
Age > 7 years, n (%)	39 (55)	2 (28.6)	
Nutritional status, n (%)			
Obesity	21(31)	2 (28.5)	0.25
Normal	38 (53.5)	7(100)	0.018
Days in PICU, median (IQR)	11 (6-114)	13(9-65)	0.46
Days from onset of symptoms to admission to PICU (IQR)	5(1-92)	6 (3-12)	0.003^b^
Skin and mucous membranes, n (%)			
Lips/tongue	16 (22.5)	1(14.3)	0.61
Hand/foot edema	32 (45.1)	2(28.5)	0.46
Conjunctival injection	23 (32.4)	0	0.09
Gastrointestinal, n(%)			
Diarrhea	54 (76.1)	1(14.3)	0.002
Vomiting	54 (76.1)	4(57.1)	0.36
Abdominal pain	51 (72)	4(57.1)	0.42
Cardiovascular, n(%)			
Shock	61 (86)	7(100)	0.98
Hypotension	55(77.5)	6(85.7)	0.6
Arrhythmia	8(11.2)	3(42.9)	0.05
**Pericarditis**	0	2(28.6)	0.007
Renal, n (%)			
Acute kidney injury	17(24)	6(85.7)	<0.001
Ferritin > 500 mcgr/dl, n (%)	32(45)	7(100)	0.012
C-reactive protein > 4 mgr/dl, n (%)	58(82)	4(57)	0.14
Dimero D > 3000 ngr/ml, n(%)	39(55)	5(71)	0.46
Respiratory support, n (%)			
NIV	68(96)	0	1.0
Conventional MV	15 (21.1)	7(100)	<0.001
HFOV	1 (1.4)	4(57.1)	<0.001
Dialysis (RRT), n(%)	3(4.2)	6(85.7)	<0.001

NIV – noninvasive ventilation. MV – mechanical ventilation. HFOV – high-frequency oscillatory ventilation. RRT – renal replacement therapy. ^a^ Fisher-exact test. ^b^Mann-Whitney U test

**Table 3:** Factors associated with mortality (by a logistic regression model)

	^**a**^	95% CI for β	P-value^**b**^	Odds Ratio (OR)^**c**^	95% CI for OR
Arrhythmia (No)^d^	1.79	(0.26, 3.32)	0.02	35.73	(1.67, 764.66)
Diarrhea (No)^e^	-2.77	(-4.89, -0.65)	0.01	0.004	(0.001, 0.272)
Ferritin^f^	-0.003	(-0.005, -0.001)	0.02	0.997	(0.994, 0.999)

Regardless of age

^a^ The numbers in this column are estimates of the coefficients using the logistic regression model for a dichotomous variable as response (in this case, Mortality which is coded as 0 if the patient survives, 0 otherwise)

^b^ Tests the null hypothesis that β is equal to 0 vs. the hypothesis that β is different from 0

^c^ The odds ratio and its CI are adjusted for Arrhythmia, Diarrhea, and Ferritin

^d^ This dichotomous variable was defined as 1 if the patient had Arrhythmia, 0 otherwise

^e^ This dichotomous variable was defined as 1 if the patient had Diarrhea, 0 otherwise

^f^ A one-unit increase in ferritin levels decreases the log of odds of survival by -0.003 units. In other words, the higher the ferritin levels, the greater the risk of mortality

#### Comparison with MIS-C in high-income countries

**Fig. 2:**
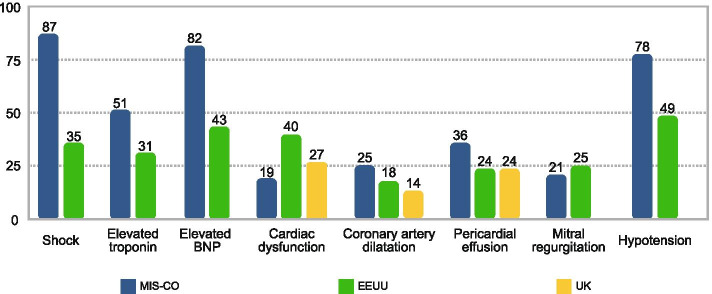
Comparison of the cardiovascular involvement in MIS-C between Colombia (MISCO STUDY), the US and the UK

**Fig. 3:**
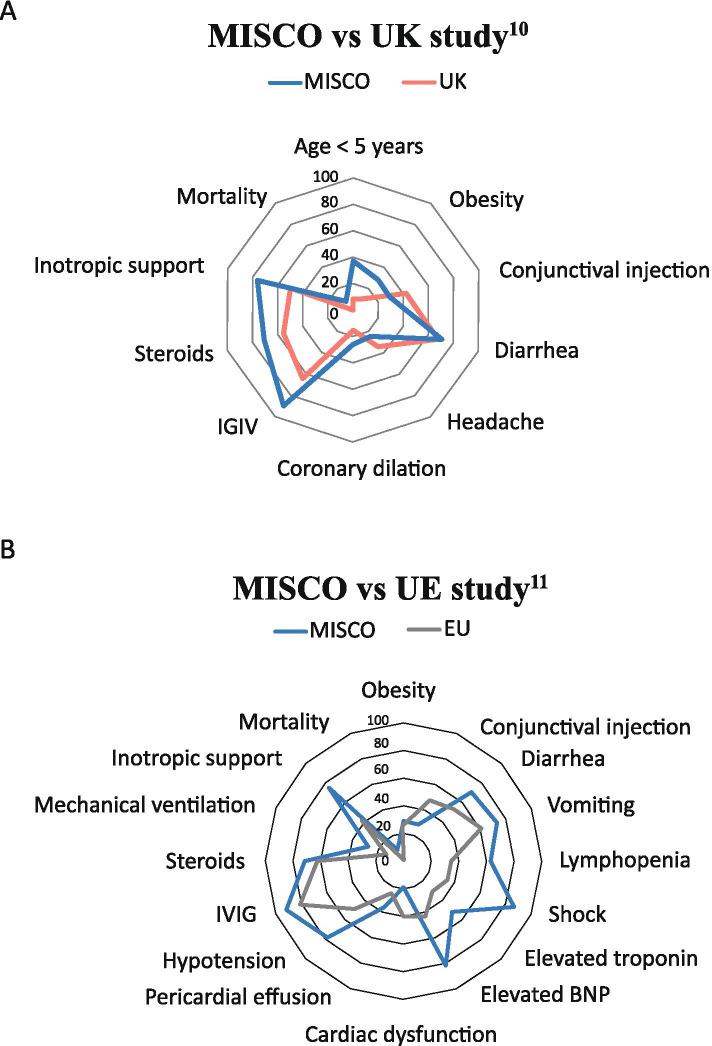
Comparison MISCO with other studies

Table 4 shows the comparison of clinical characteristics between the subjects in our study and those reported in the US study by Godfred-Cato et al. [8] between March and July 2020, and the UK study by Swann et al. [12] between January and July 2020. With regard to sex, there were no statistically significant differences between the three studies. Obesity was more frequent in our study (29.5 % vs. 10 %; p=0.008) compared to the data reported in the UK. In comparison to the US study, lymphadenopathy was more frequent in our population (23 % vs. 13 %; p=0.002), as was diarrhea (70.5 % vs. 53 %; p=0.001), vomiting (74 % vs. 62 %; p=0.03) and lymphopenia (64 % vs. 35 %; p=0.001). With regard to the treatment received, in our study there was more frequent use of IVIG (91 % vs. 81 %; p=0.001), steroids (70.5 % vs. 63 %; p=0.03) and vasopressor support (76 % vs. 42 %; p=0.001). Likewise, mechanical ventilation was needed more often (28 % vs. 13 %; p=0.001) and there was greater mortality (9 % vs. 1.8 %; p=0.001). Figures 2 and 3 and Table 4.

**Table 4:** Comparison of Colombian data with other studies

	MISCO study n (%)	MIS-C USA^11^ n (%)	P value^a^	MIS-C UK^10^ n (%)	P value^a^
Number of patients	78	570		52	
Patients in PICU	78 (100)	364 (63.9)		38 (73)	
Age in years (median, IQR)	7 (1 - 11)	8 (4 - 12)		4.6 (0.3 - 13.7)	
Age		N/R			
< 5 years	29/78 (37)			5/52 (10)	0.004
≥ 5 years	49/78 (63)			47/52 (90)	
Sex					
Male	46/78 (59)	316/570 (55)	0.55	31/52 (60)	0.94
Female	32/78 (41)	254/570 (45)		21/52 (40)	
Obesity	23/78 (29.5)	146/570 (26)	0.46	5/51 (10)	0.008
Symptoms of KD					
Fever	78/78 (100)		NA	52/52 (100)	NA
Changes in the mouth and mucous membranes	17/78 (22)	201/570 (35)	0.018		
Lymphadenopathy	18/78 (23)	76/570 (13)	0.02	9/46 (19.5)	0.6
Conjunctival injection	23/78 (29.5)	276/570 (48)	0.001	20/48 (42)	0.16
Other symptoms					
Diarrhea	55/78 (70.5)	303/570 (53)	0.001	32/49 (65)	0.53
Abdominal pain	55/78 (70.5)	353/570 (62)	0.14	34/50 (68)	0.76
Nausea/vomiting	58/78 (74)	352/570 (62)	0.03	38/50 (76)	0.83
Headache	17/78 (22)	186/570 (33)	0.052	16/47 (34)	0.13
Labs					
Lymphopenia	50/78 (64)	202/570 (35)	0.001		
Thrombocytopenia	28/78 (36)	176/570 (31)	0.37	16/50 (32)	0.65
Cardiac complications					
Shock	68/78 (87%)	202/570 (35)	0.001		
Elevated troponin	38/75 (51%)	176/570 (30.9)	0.006		
Elevated BNP	27/33 (82%)	246/570 (43)	0.001		
Cardiac dysfunction	14/75 (19)	207/510 (40.6)	0.002		
Coronary dilation	19/75 (25)	95/510 (18.6)	0.17	10/37 (27)	0.31
Pericardial effusion	27/75 (36)	122/510 (23.9)	0.02		0.15
Mitral regurgitation	16/75 (21)	130/510 (25.5)	0.43	5/37 (13.5)	0.21
Hypotension	61/78 (78)	282/570 (49.5)	0.001	9/37 (24)	
IVIG	71/78 (91)	424/570 (81)	0.001	28/43 (65)	0.004
Steroids	55/78 (70.5)	331/570 (63)	0.03	24/44 (55)	0.07
Mechanical ventilation	22/78 (28)	69/570 (13)	0.001	14/52 (27)	0.87
High-flow cannula	24/78 (31)		NA	23/52 (44)	0.11
Inotropic support	59/78 (76)	221/570 (42)	0.001	25/49 (51)	0.004
Death	7/78 (9)	10/570 (1.8)	0.001	0/51	NA

^a^ Chi square test or Fisher’s exact test, as applicable. KD: Kawasaki disease. N/R Not reported

## Discussion

In this study, we described 78 children under the age of 18 living in a middle-income country, who met the criteria for MIS-C associated with SARS-CoV-2. Due to the severity of their clinical condition, these patients needed to be transferred to the pediatric intensive care unit. We observed that the affected children were younger and had more obesity, cardiovascular and gastrointestinal symptoms at the time of presentation than those in studies published in the US [8] and UK [12]. In addition, these patients required vasoactive support and mechanical ventilation more often and had a greater mortality rate compared to high-income countries.

In our study, 67 % had a positive RT-PCR for SARS-CoV-2 at diagnosis, which is a higher frequency when compared to what has been observed in other studies [8]. 33 % of our patients had serological evidence of SARS-CoV-2 infection with a negative RT-PCR. Likewise, the behavior of MIS-C in terms of frequency and severity also occurred 4-6 weeks after the peak in adults, as has been described.

Feldstein et al. [9] reported that the typical population affected by MIS-C is that of school-age children, as we saw in our study. However, 18 % of our population was under one year old and met the WHO criteria for MIS-C. These younger patients did not have more cardiovascular complications or greater mortality than the older children and had a very similar clinical course.

Similarly, it has been reported that MIS-C may occur more frequently in obese patients. The studies report a frequency ranging from 10 to 25 % of cases having overweight and obesity [8, 9, 12]. We found this comorbidity in 37 % of our patients. In middle and low-income countries, malnutrition related to poverty and scarce resources is increasingly found. According to WHO, 11.8 % of children who live in middle-income countries have some type of malnutrition. Of these, 9.8 % were overweight and obese, which accounted for approximately 41 million children worldwide [13, 14] in 2016. Diets based on complex carbohydrates and few proteins, along with more sedentary habits, are some of the explanations found by WHO to explain this phenomenon in countries with limited resources [13]. In children affected by MIS-C, obesity has been associated with greater hyperinflammatory response and endothelial, adipocyte, and macrophage activation, which explains some biomarkers’ elevation in this disease [15]. Endothelial activation may lead to vasoconstriction, prothrombotic states and the release of anti-mitogenic mediators [16–22]. The association between obesity and the severity of endothelial activation and dysfunction in critically ill children is widely studied, particularly with the more significant oxidative stress and insulin resistance which may occur in these patients [23, 24].

In this regard, SARS-CoV-2 has been considered by some to be a systemic endothelitis [17–19], endothelial glycocalyx damage [24]. Consiglio et al. [15], in a study of healthy children, children with COVID-19 and controls with Kawasaki disease and MIS-C, analyzed immune system cells, cytokines, and antibodies, finding that the inflammatory response in MIS-C mediated by the cytokine storm may be similar to that in Kawasaki disease, but with a few differential factors. There were differences in the T cells’ response, IL-17 A, and a few other biomarkers associated with arterial injury in MIS-C. In particular, they found antibodies directed against some structural endothelial glycoproteins, such as endoglin, which suggests that these autoantibodies could be a research focus to determine targeted treatment for children with MIS-C [15].

Furthermore, in our study, we found shock, elevated troponin and proBNP, and myocardial dysfunction more often than in high-income countries [8, 12]. We believe that several factors may explain this situation. In addition to the inflammatory response and endothelial activation seen, it is possible that healthcare system factors, such as rapid access to care, may have contributed to the higher frequency of cardiovascular involvement. Moreover, the lack of recognition may have contributed to the progression of the disease, with more frequent hemodynamic involvement. However, the frequency of coronary dilation and other cardiac involvement was similar to what has been seen in other countries.

Interestingly, in our study, 29 % of the critically ill children were found to have kidney involvement on admission. The study by Feldstein et al. [9] reported 17 % of this involvement in SARS-CoV-2 infection. Although the pathophysiological mechanisms are unknown, we believe that in our study, delayed consultation, the lack of early recognition, the more significant inflammatory response, and frequent cardiovascular involvement, among others, may explain this greater frequency of kidney injury. Adults report 20 % of kidney involvement in COVID-19, which may reach 50 % in ICU patients, with a frequent need for continuous renal replacement therapy [25, 26]. In children with MIS-C, LiptonM et al. [25] described 26 patients with acute kidney injury (AKI). This group found that renal involvement was associated with a greater elevation of CRP, ferritin, and procalcitonin, with greater lymphopenia and left ventricular dysfunction. It is suggested that the risk factors for acute kidney injury in children with MIS-C are greater age, greater inflammatory involvement, and ventricular dysfunction [25, 27, 28]. In our study, we found high CRP and ferritin, indicative of a significant inflammatory response, along with frequent systolic myocardial dysfunction, which might explain, in part, the frequency of AKI seen.

In our study, there was much higher mortality (9 % vs. 1 %) than that described in high-income countries [4–7, 12]. We believe that several factors may contribute to these worse outcomes. Limited access to healthcare services [2], PICU availability, delayed consultation, greater severity of the myocardial involvement, arrhythmia, and shock at the time of admission, along with lack of recognition of the disease, may have contributed to these worse outcomes. Other factors such as environment, lifestyle, and availability of resources for timely health care must be considered in children with MIS-C. In a recent multicenter case-control study, children from minority racial or ethnic backgrounds were disproportionately at risk for developing MIS-C [29]. We believe that in low and middle income countries, we should have a high level of suspicion at the primary level of care. In order to make a rapid and adequate diagnosis, the early initiation of anti-inflammatory treatment and anticoagulation when indicated can significantly improve patient outcomes.

We consider that our study has several limitations. First, not all the pediatric intensive care units in the country were included, which may have led to an under-reporting of critical MIS-C cases. However, the participating centers are considered referral centers for most nearby towns, which capture a significant proportion of the pediatric population in Colombia. In addition, children with MIS-C who were not transferred to intensive care were not included because our objective was to analyze the characteristics of critically ill children with MIS-C. This decision may lead to an information bias, as only data from the most severely ill children is available. Likewise, data on inflammatory biomarkers, among others, and the progression of all the children affected by COVID-19 nationwide, were not available to make up a comparison group with less severe disease. Although we know that MIS-C cases are increasingly described worldwide, we decided to make a comparison with data reported up to June 2020 in the US and the UK (with a smaller study). More data from more extensive studies in these countries might show smaller differences. Still, we consider that in countries with limited resources, the socioeconomic and environmental factors may contribute to the observed outcomes. Finally, during the study period, variants of SARS-CoV-2 had not been reported in the country. We believe it is important to keep a close watch on the different viral variants in these patients´ records and whether they have a different effect in children in terms of severity.

## Conclusions

Multisystem Inflammatory Syndrome in Children associated with SARS-CoV-2 in critically ill children living in a middle-income country has some clinical, laboratory, and echocardiographic characteristics, similar to those described in high-income countries. Inflammatory response and cardiovascular involvement were conditions that added to the difficulties in accessing the healthcare system in countries with limited resources, could explain the greater mortality seen in these children. Prospective studies are needed to compare the different strategies of early recognition, availability of resources and type of treatments used to better understand the differences found and the MIS-C outcomes in countries with limited resources compared to high-income countries.

## Supplementary Information

**Additional file 1.** Lab results.

**Additional file 2.** Estimated survival curve, based on a Cox model. The absence of arrhythmia has an HR=0.031, 95%CI=0.002-0.408, p=0.031; indicating that patients with arrhythmia tend to die faster than patients without it.

**Additional file 3.**

## Data Availability

Datasets used or analyzed during the current study are available from the corresponding author on reasonable request.

## Abbreviations

MIS-C: multisystem inflammatory syndrome in children asocciated with COVID-19
PIMS-TS: paediatric Multisystem inflammatory syndrome – temporally associated with SARS-COV2
PICU: pediatric intensive care unit
RT-PCR: real time – polymerase chain reaction
Pro-BNP: proB-natriuretic peptide
VIS: vasoactive and inotropic score
PCT: procalcitonin
CRP: C –reactive protein
MV: mechanical ventilation
HFOV: high-frecuency oscillatory ventilation
AKI: acute kidney injury
HRs: hazard ratios
CIs: confidence intervals
