# Supplementary material for: Mortality and clinical characteristics of multisystem inflammatory syndrome in children (MIS-C) associated with covid-19 in critically ill patients: an observational multicenter study (MISCO study)
Source: BMC Pediatr. 2021 Nov 18;21:516. doi: 10.1186/s12887-021-02974-9 (PMC8600488; doi:10.1186/s12887-021-02974-9)
Supplement: Supplementary file 1 — Additional file 1. Lab results. [file 12887_2021_2974_MOESM1_ESM.docx]

**Additional file 1. Lab results**

| Lab test | n | Admission | n | 24 hours | n | 48 hours | P value ^§^ |
| --- | --- | --- | --- | --- | --- | --- | --- |
| CRP (mg/dL) | 78 | 12.7 (5.9-20.5) | 67 | 11.6 (3.9-19.5) | 66 | 8.2  (2.8 - 15) | 0.003 |
| ESR (mm/h)* | 63 | 45 (25-58) |  |  |  |  |  |
| Procalcitonin (ng/mL) | 50 | 1.37 (0.88-6.18) |  |  |  |  |  |
| Leukocytes (cells/𝜇l) | 78 | 9,800 (7,530-16,202) |  |  |  |  |  |
| D-dimer (ng/mL) | 75 | 3,553 (2,090-6,500) | 46 | 3,820  (1,850-7,399) | 54 | 3,442  (1,640-6,135) | 0.02 |
| Ferritin (ng/mL) | 78 | 500 (308-950) | 63 | 450  (205-932) | 54 | 503  (317-946) | 0.47 |
| Fibrinogen (mg/dL) | 76 | 342 (218-539) |  |  |  |  |  |
| LDH* | 78 | 332 (255-567) |  |  |  |  |  |
| Troponin (ng/mL) | 75 | 0.1 (0.03-0.31) | 43 | 0.08  (0.03-0.31) | 34 | 0.06  (0.01 - 0.22) | 0.012 |
| ProBNP (pg/mL) | 33 | 1,500 (630-4,507) |  |  |  |  |  |
| Albumin ** | 76 | 2.3 (2.1-2.7) |  |  |  |  |  |

CRP – C-reactive protein. ESR – Erythrocyte sedimentation rate. Values expressed as Median (IQR). *Highest value. **Lowest value. § Friedman test.
