# Supplementary material for: Mortality and clinical characteristics of multisystem inflammatory syndrome in children (MIS-C) associated with covid-19 in critically ill patients: an observational multicenter study (MISCO study)
Source: BMC Pediatr. 2021 Nov 18;21:516. doi: 10.1186/s12887-021-02974-9 (PMC8600488; doi:10.1186/s12887-021-02974-9)
Supplement: Supplementary file 2 — Additional file 2. Estimated survival curve, based on a Cox model. The absence of arrhythmia has an HR=0.031, 95%CI=0.002-0.408, p=0.031; indicating that patients with arrhythmia tend to die faster than patients without it. [file 12887_2021_2974_MOESM2_ESM.docx]

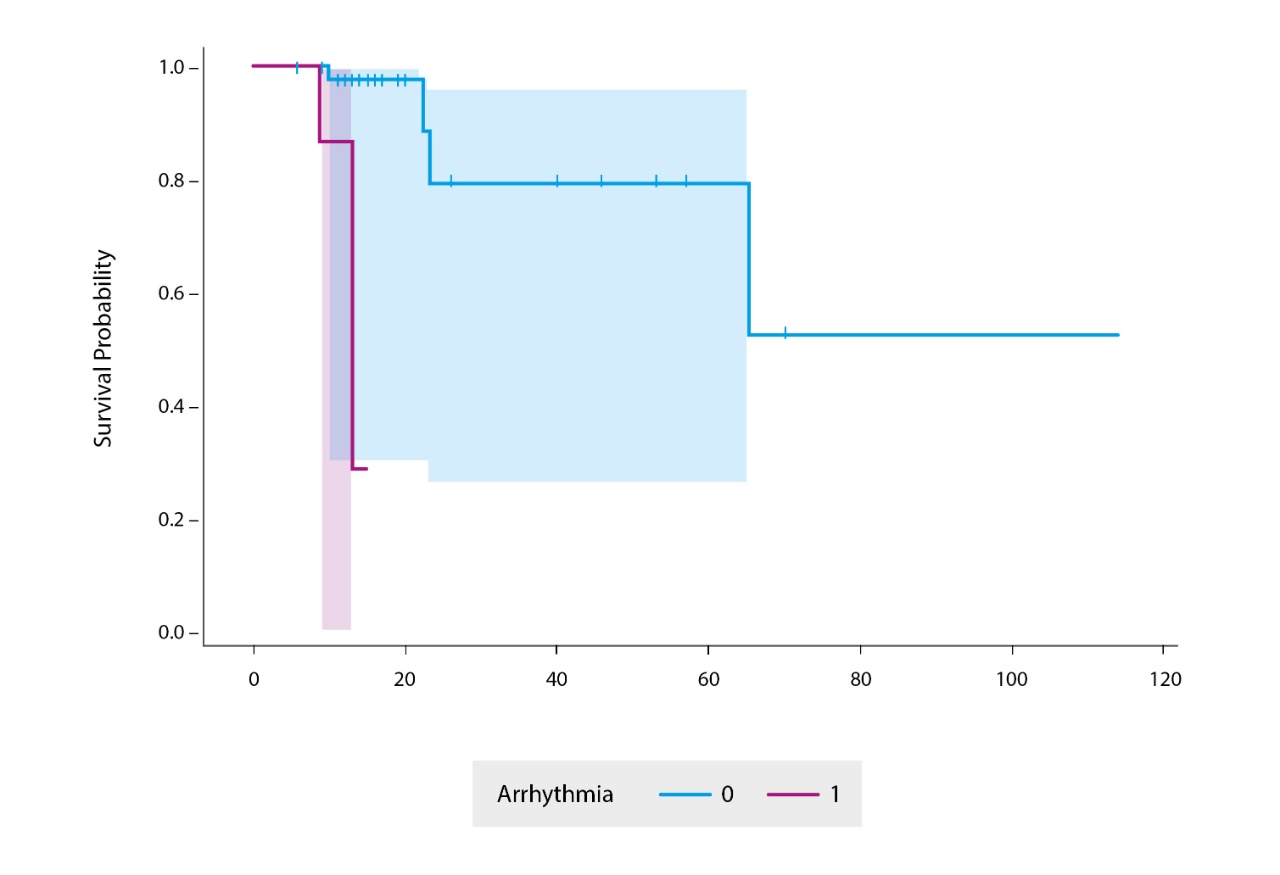


**Additional file 2** . Estimated survival curve, based on a Cox model. The absence of arrhythmia has an HR=0.031, 95%CI=0.002-0.408, p=0.031; indicating that patients with arrhythmia tend to die faster than patients without it.
