# Supplementary figures and images for: Mortality and clinical characteristics of multisystem inflammatory syndrome in children (MIS-C) associated with covid-19 in critically ill patients: an observational multicenter study (MISCO study)
Source: BMC Pediatr. 2021 Nov 18;21:516. doi: 10.1186/s12887-021-02974-9 (PMC8600488; doi:10.1186/s12887-021-02974-9)

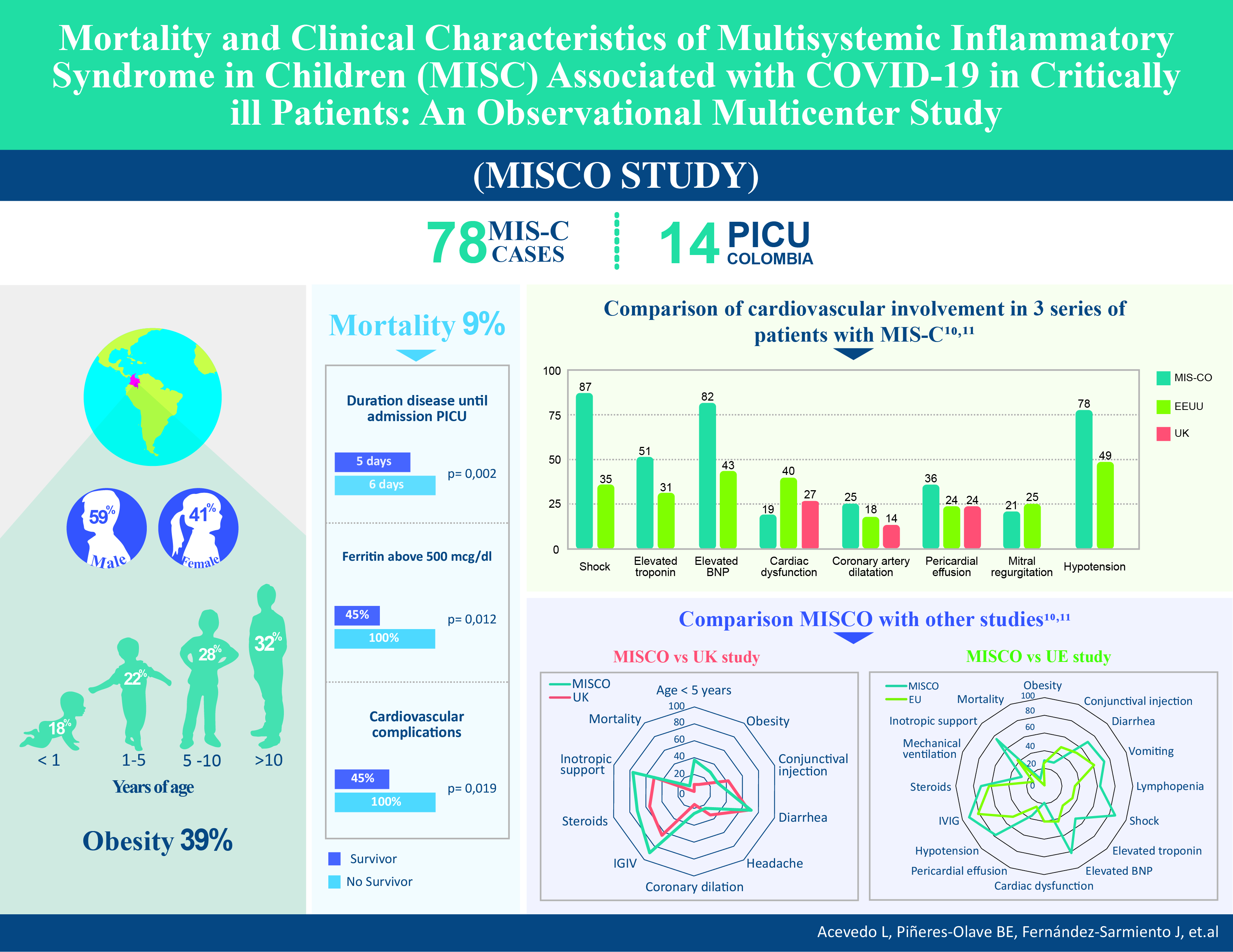

Supplement: Supplementary file 3 — Additional file 3. [file 12887_2021_2974_MOESM3_ESM.tif]
